# Diurnal rhythms in the human urine metabolome during sleep and total sleep deprivation

**DOI:** 10.1038/srep14843

**Published:** 2015-10-09

**Authors:** Guro F. Giskeødegård, Sarah K. Davies, Victoria L. Revell, Hector Keun, Debra J. Skene

**Affiliations:** 1 Department of Circulation and Medical Imaging, Norwegian University of Science and Technology, Trondheim, Norway; 2 St. Olavs Hospital, Trondheim University Hospital, Trondheim, Norway; 3 Chronobiology, Faculty of Health and Medical Sciences, University of Surrey, Guildford, Surrey GU2 7XH, UK; 4 Department of Surgery and Cancer, Imperial College London, London, SW7 2AZ, UK

## Abstract

Understanding how metabolite levels change over the 24 hour day is of crucial importance for clinical and epidemiological studies. Additionally, the association between sleep deprivation and metabolic disorders such as diabetes and obesity requires investigation into the links between sleep and metabolism. Here, we characterise time-of-day variation and the effects of sleep deprivation on urinary metabolite profiles. Healthy male participants (n = 15) completed an in-laboratory study comprising one 24 h sleep/wake cycle prior to 24 h of continual wakefulness under highly controlled environmental conditions. Urine samples were collected over set 2–8 h intervals and analysed by ^1^H NMR spectroscopy. Significant changes were observed with respect to both time of day and sleep deprivation. Of 32 identified metabolites, 7 (22%) exhibited cosine rhythmicity over at least one 24 h period; 5 exhibiting a cosine rhythm on both days. Eight metabolites significantly increased during sleep deprivation compared with sleep (taurine, formate, citrate, 3-indoxyl sulfate, carnitine, 3-hydroxyisobutyrate, TMAO and acetate) and 8 significantly decreased (dimethylamine, 4-DTA, creatinine, ascorbate, 2-hydroxyisobutyrate, allantoin, 4-DEA, 4-hydroxyphenylacetate). These data indicate that sampling time, the presence or absence of sleep and the response to sleep deprivation are highly relevant when identifying biomarkers in urinary metabolic profiling studies.

Metabolomics is the study of metabolites; small-molecular compounds that are intermediates or end-products of metabolism. Urine is a commonly used biofluid for metabolomics studies as it is non-invasive and easily acquired, and changes in urinary metabolic profiles have been linked to different disease states, such as congenital defects in metabolism^1^, hypertension^2^ and poor development in pregnancy^3^. While a number of studies have shown that urinary metabolites vary with time of day in humans^4^,^5^,^6^, low sampling rate/24 h and confounding factors (light/dark environment; sleep/wake cycle; variable meal times) make conclusions difficult. We thus aimed to characterize urinary metabolite rhythms (6 set time periods/24 h) in healthy subjects across the 24 h day (time-of-day variation) in controlled conditions, and to investigate the impact of sleep and sleep deprivation.

There have been multiple studies demonstrating that gene expression is affected by disrupted sleep^7^,^8^,^9^. However, as it becomes increasingly evident that chronic sleep restriction, sleep deprivation and circadian misalignment are associated with cardiovascular disease (CVD), obesity and multiple metabolic disorders including diabetes and insulin resistance^10^,^11^,^12^,^13^, there is a clear need to characterize the connection between sleep and metabolism. We have recently described how the plasma metabolome varies with time of day and under one night of acute total sleep deprivation, mainly lipids and acylcarnitines increasing during sleep deprivation^14^. Moreover, potential metabolite markers of reduced sleep duration (chronic partial repeated sleep restriction) have recently been identified, two of which seem to be conserved between rats and humans (oxalic acid and one diacylglycerol^15^) with the majority of markers of restricted sleep in both species being lipids.

The aim of this study was to characterize time-of-day variation and the effect of sleep deprivation on metabolites in another readily accessible biofluid (urine) using a different analytical platform well suited to large scale urinary studies (NMR spectroscopy). Describing how metabolites change during the 24 h day under sleep/wake and continual wakefulness would be beneficial for optimizing urine sample selection procedures for clinical and epidemiological metabolomics and exposome studies, in order to minimize unrelated variation in metabolite levels resulting from food intake, time of day, or an individual’s sleep status. Furthermore we present the first controlled study of the impact of total sleep deprivation on the NMR-detectable urinary metabolome.

## Methods

### Study participants and sample collection

**Figure 1:**
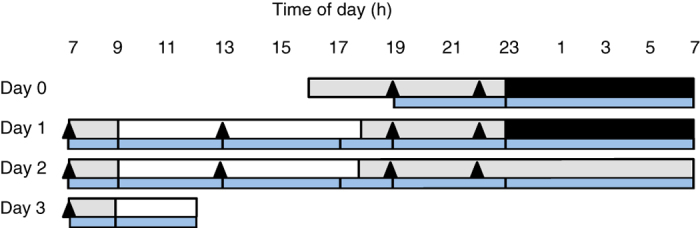
A schematic of the in-laboratory protocol. The in-laboratory session consisted of an adaptation night (day 0) followed by a 24 h wake/sleep cycle and a 24 h wake/wake cycle. White sections indicate wake periods (100 lux, free to move); grey sections indicate wakefulness in a semi-recumbent position in dim light (<5 lux); black sections indicate sleep periods in a supine position in the dark (0 lux with eye masks). Standardized meals are represented by triangles; blue sections show the set urine collection time intervals. The midpoint time of each urine collection was used for statistical analyses. Modified from Ackermann *et al.* [16] with permission.

The study was approved by the University of Surrey Ethics Committee (EC/2011/127/FHMS) and conducted according to the Declaration of Helsinki. All participants provided written informed consent prior to participating in the study. Healthy male volunteers (n = 15, age 23.7 ± 5.4 years (mean ± SD)) were kept in controlled laboratory conditions with respect to environmental light/dark, sleep, meals and posture during a 24 h wake/sleep cycle (8 h sleep period 23:00–07:00 h), followed by 24 h of wakefulness (total sleep deprivation). The screening process and study protocol has been described in detail by Davies *et al.*^14^ and Ackermann *et al.*^16^ In brief, all potential participants underwent a medical screening, including completing a range of sleep and health questionnaires, and were healthy non-smokers, not taking medication and had no self-reported sleep problems. For one week prior to the in-laboratory session, study participants maintained a regular sleep/wake schedule (sleep 23:00–07:00 h), confirmed by wrist actigraphy (Actiwatch-L, Cambridge Neurotechnology Ltd., UK), sleep diaries, and calling a time-stamped voicemail before going to sleep and upon waking. The participants were requested not to consume alcohol or caffeine, use non-steroidal anti-inflammatory drugs, perform heavy exercise, or be exposed to bright light in the evenings and nights for 72 hours before the in-laboratory session to ensure a stable circadian phase and minimize sleep deprivation prior to entry into the laboratory study. The in-laboratory session (Fig. 1) consisted of an adaptation night followed by a 24 h wake/sleep cycle (day 1) and a 24 h wake/wake cycle (day 2), thus the study participants spent a total of 72 h in the laboratory. Standardized meals were provided at 07:00, 13:00, 19:00 and 22:00 h. Between 09:00–18:00 h the participants were allowed to move freely in ~100 lux; between 18:00–23:00 h and 07:00–09:00 h the participants were in a semi-recumbent position in dim environmental lighting (<5 lux), and during the night period (23:00–07:00 h) the participants were in recumbent position on day 1 (0 lux) with an opportunity to sleep and semi-recumbent and awake on day 2 (<5 lux).

During the in-laboratory session, pooled total volume urine samples were collected at set intervals of 2–4 h during the day time and 8 h overnight (sampling intervals: 19:00–23:00, 23:00–07:00, 07:00–09:00, 09:00–13:00 (09:00–12:00 on day 3), 13:00–17:00, 17:00–19:00 h, Fig. 1). The subjects went to the toilet to urinate between 09:00–18:00 h, while they used bedpans for urinating between 18:00–09:00 h. The samples were stored at −80 °C within 1 hour of the end of the collection interval. The maximum and minimum length of time the urine was at room temperature was 5.3–0 h and 6.3–1.7 h during the sleep and sleep deprivation period, respectively.

### NMR analysis

Samples were prepared for analysis essentially as described by Dona *et al.*^17^. Urine samples were thawed at room temperature, and centrifuged at 1200 g at 4 °C for 10 minutes. The supernatant (540 μL) was mixed with buffer (60 μL) (pH 7.4; 1.5 mM KH_2_PO_4_, 0.1% TSP, and 2 mM NaN_3_ in D_2_O) in 5 mm NMR tubes using a liquid handler robot. ^1^H one-dimensional Standard Nuclear Overhauser Effect spectroscopy (1D-NOESY) (noesygppr1d; Bruker) spectra with water pre-saturation and 2D J-resolved (JRES) spectra were acquired at 300 K on a Bruker Avance 600 MHz spectrometer (Bruker Biospin GmbH, Germany). Experiments were fully automated using the SampleJet in combination with Icon-NMR on TopSpin 3.1 software (Bruker Biospin), keeping the samples cooled (4 °C) prior to analysis.

### Data preprocessing and multivariate data analysis

The resulting FIDs of the 1D NOESY spectra were automatically Fourier transformed with an exponential line broadening of 0.3 Hz, phased, and baseline corrected in Topspin. Preprocessed 1D NOESY spectra were transferred into Matlab R2013b (The Mathworks, Inc., USA) and referenced to the TSP peak at 0 ppm, before peak alignment by recursive segment-wise peak alignment (RSPA)^18^. The spectrum with the highest correlation to the other spectra was used as the alignment reference. Five low quality spectra with poor water suppression and/or shim (TSP halfwidth >1.3 Hz) were removed from further analyses, leaving a data set of 586 spectra. The spectral data between 0.6 and 9.3 ppm were extracted for multivariate analysis, after removal of the residual water (4.5–5.2 ppm) and urea (5.5–6.1 ppm) regions. Prior to multivariate analysis, the spectra were binned (bin size 0.01 ppm) and normalised by probabilistic quotient normalisation (PQN)^19^. Multivariate analysis was performed on mean-centered data by orthogonalized partial least squares discriminant analysis (OPLS-DA) validated by leave-one-subject-out cross validation and permutation testing (n = 1000, significance for p_perm_ ≤ 0.05). Multivariate analyses were performed in Matlab using PLS_toolbox 7.8.2 (Eigenvector Research, Inc., USA).

### Identification and relative quantification of metabolites

Metabolites were assigned using Chenomx NMR suite 7.7 (Chenomx Inc., Alberta, Canada), the Human Metabolome Database^20^, and/or literature values (4-deoxyerythronic acid (4-DEA)^21^,^22^; 4-deoxythreonic acid (4-DTA)^22^; P-cresol sulfate^23^,^24^); and by 2D JRES spectroscopy. Statistical Total Correlation Spectroscopy (STOCSY)^25^ was applied for some metabolites for identification of correlating peaks in the spectrum. Individual metabolite peaks were integrated and quantified relative to the “Electronic REference To access *In vivo* Concentrations” (ERETIC) signal^26^. The area under the ERETIC signal corresponded to 10 mM of proton signal in this study. For metabolites with more than one resonance, either the mean or the resonance in a non-overlapping region of the spectrum was used. The resulting data were normalised by PQN^19^ to correct for differences in urine concentrations. PQN estimates the most probable dilution factor of a sample and scales the sample accordingly. The most probable dilution factor was calculated as the median quotient between the variables of a sample and a reference sample after integral normalisation. The median sample was used as a reference sample.

### Statistical analysis of individual metabolites

Differences in individual metabolite levels at different time points were examined by linear mixed models analysis (LMM). LMM provides great flexibility and allows for missing values in paired analyses, thus all available data are utilized. LMM was also used for modelling time related trends for comparison of metabolite levels between day 1 and 2. LMM was performed in R (version 3.0.3, R Foundation for Statistical Computing) using the NLME package. The data were log transformed prior to LMM analysis in order to obtain normally distributed residuals. Benjamini-Hochberg correction for multiple testing was performed, and q values ≤ 0.05 were considered significant. Time-of-day variations of identified metabolites were examined by principal component analysis (PCA) of autoscaled metabolite levels (PLS_toolbox).

Correlations between metabolites were examined by Spearman’s rank correlations. The results are presented in a heatmap ordered by hierarchical clustering (HC). HC was performed in Matlab using Spearman’s correlation as a similarity measure and average linkage for grouping of clusters.

### Cosinor analysis

Cosinor analysis was performed on the mean z-score values at each time point (midpoint of each urine collection interval was used; z-scores were calculated across 48 h) for each metabolite profile for both day 1 (wake/sleep condition, 11:00–11:00 h) and day 2 (sleep deprivation condition, 11:00–10:30 h). The fit of the data to a cosine function (y = a + A cos wt + B sin wt, with the assumption that all subjects were entrained to a 24 hour rhythm, so that w = 2 pi/24 hours^(−1)) was compared with fit to a y = c model, generating a p value in each case (p values were adjusted to take account of multiple testing using the Benjamini-Hochberg method). Peak time (acrophase) and significance of cosine fit (p < 0.05) were determined in each case.

## Results

**Figure 2:**
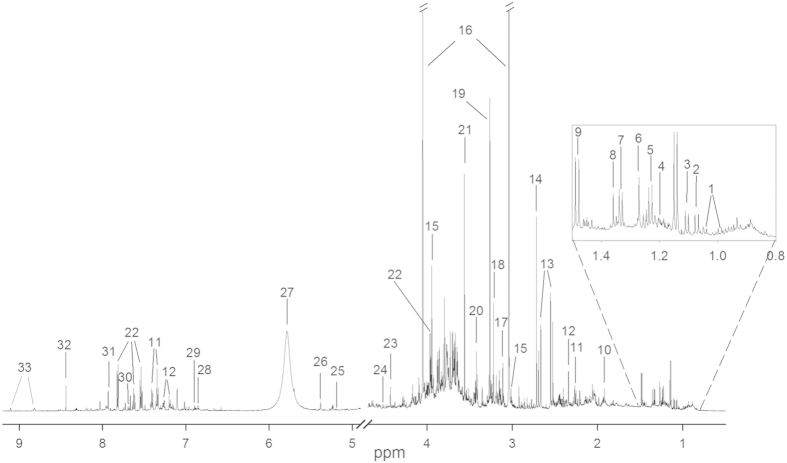
A typical urine ^1^H NMR spectrum with identified metabolites. The metabolites are numbered accordingly: 1: valine, 2: 3-hydroxyisobutyrate, 3: 4-deoxyerythronic acid (4-DEA), 4: 3-aminoisobutyrate, 5: 4-deoxythreonic acid (4-DTA), 6: 3-hydroxyisovalerate, 7: threonine/lactate, 8: 2/alfa-hydroxyisobutyrate, 9: alanine, 10: acetate, 11: phenylacetylglutamine, 12: p-cresol sulfate, 13: citrate, 14: dimethylamine, 15: creatine, 16: creatinine, 17: proline betaine, 18: carnitine, 19: trimethylamine-N-oxide (TMAO), 20: taurine, 21: glycine, 22: hippurate, 23: trigonelline, 24: ascorbate, 25: xylose, 26: allantoin, 27: urea*, 28: 4-hydroxyphenylacetate, 29: tyrosine, 30: 3-indoxyl sulfate, 31: histidine, 32: formate, 33: trigonelline, *urea peak was not quantified.

Figure 2 shows a representative 1D NOESY NMR spectrum of a urine sample from one of the study participants with the most abundant metabolites assigned. A total of 32 metabolites were successfully identified from the spectra with relative quantification calculated using the ERETIC signal (Table 1). The doublet signals from lactate (lac) and threonine (thr) at approximately 1.33 ppm are overlapping, and these metabolites were integrated as one common peak (thr+lac). The raw values (mM) for all metabolites (n = 32) measured for each participant (n = 15) at each urine collection (ml) is presented in Supplementary Table S1.

### Metabolic changes during and after sleep deprivation

**Figure 3:**
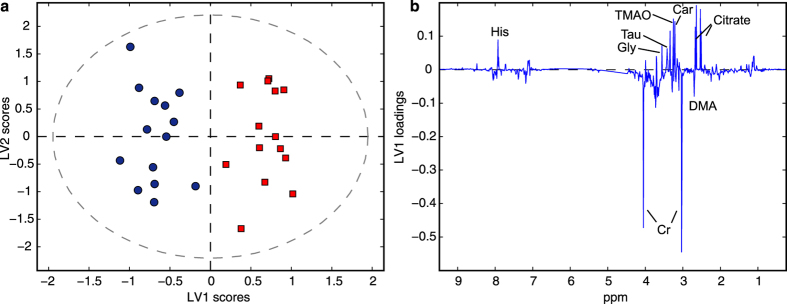
Metabolic differences during a night of sleep deprivation compared to a night of sleep (a) OPLS-DA scores and (b) loadings separating NMR spectra of urine samples from a night of sleep deprivation and a night of sleep. The OPLS-DA model explains 54.1% and 89.5% of x-and y-variation, respectively. Car: carnitine, Cr: creatinine, DMA: dimethylamine, Gly: glycine, His: histidine, Tau: taurine.

OPLS-DA of the urine NMR spectra revealed significant changes in the urinary metabolic profiles during a night of sleep deprivation compared to a night of sleep (23:00–07:00 h) with classification error, sensitivity, and specificity of 7.0%, 92.9% and 92.9%, respectively (p_perm_ < 0.001) (Fig. 3). The loading plot (Fig. 3b) shows increased concentrations of glycine, taurine, trimethylamine-N-oxide (TMAO), carnitine and citrate, and decreased concentrations of creatinine (mean concentration of creatinine over time is shown in Supplementary Fig. S1) and dimethylamine during sleep deprivation. Except for glycine (q = 0.15), these findings were also significant in linear mixed models (LMM) analyses of the identified metabolites (Table 1). In total, 16 identified metabolites had significantly changed concentrations during sleep deprivation, with 8 metabolites increasing and 8 metabolites decreasing compared to during sleep (Table 1).

The concentration of five metabolites (4-DTA, taurine, 3-indoxyl sulfate, glycine, and tyrosine) were significantly different by LMM analysis between the first morning collection period following sleep (07:00–09:00 h) and the equivalent period following sleep deprivation (q < 0.05). An OPLS-DA classification model of the spectral data from the first morning collection period resulted in classification error, sensitivity and specificity of 14.8%, 84.6% and 85.7%, respectively (p_perm_ = 0.001). These metabolic differences, however, were not present in the second morning collection intervals (09:00–13:00 h) following sleep and sleep deprivation as evidenced by insignificant OPLS-DA classification results (p_perm_ = 0.10) for this time period.

LMM analysis of individual metabolites showed significant differences (q = 0.013) in taurine levels in the second morning collection interval (09:00–13:00 h) in day 1 vs day 2, however taurine levels were also significantly different between the two day time periods (07:00–23:00 h) prior to sleep deprivation (LMM of day time period day 1 vs day 2, q = 2.1e-05). The increased taurine observed over the full study protocol is a confounding factor in a sleep/sleep deprivation comparison. This trend of increased taurine concentration is also visible in a plot of mean taurine concentrations over time (Supplementary Fig. S1). Analysis of individual time points showed a significant difference in taurine levels at time interval 19:00–23:00 h before the nights of sleep and sleep deprivation (q = 0.0063), but no significant changes in earlier time periods. In order to baseline correct for this linear increase in taurine levels over time, the difference in taurine levels between the time interval of interest and the last time interval before sleep or sleep deprivation (19:00–23:00 h) was used as input for statistical analysis by LMM. Baseline corrected taurine levels remained significantly different between the night of sleep deprivation and a night of sleep (p = 0.0082, q = 0.0175), but not between the first morning 2 h intervals (p = 0.2274, q = 0.3751) nor the second morning intervals (p = 0.9163, q = 0.949) following sleep/sleep deprivation.

### Urinary metabolic time-of-day variation

**Figure 4:**
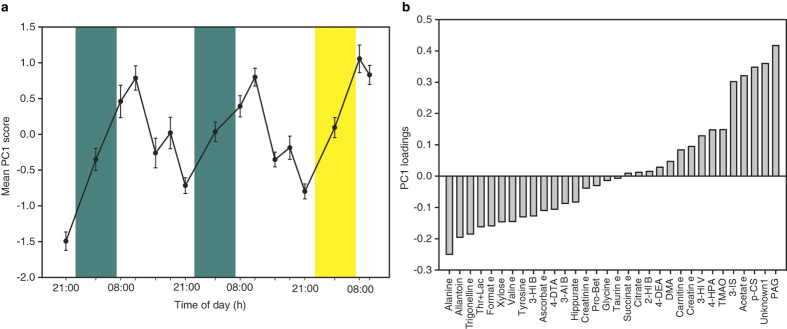
Time-of-day variations in urinary metabolites. (**a**) Mean (±SEM) PC1 scores (across all 15 individuals) from a PCA of all identified metabolites (n = 32), plotted against time, and (**b**) the corresponding PC1 loading. The individual time points are the midpoint times from intervals of pooled urine samples obtained sequentially (2–8 h) across the 60 h study protocol. Green areas, sleep period; yellow area, sleep deprivation night. 2-HIB: 2-hydroxyisobutyrate, 3-AIB: 3-aminoisobutyrate, 3-HIV: 3-hydroxyisovalerate, 3-IS: 3-indoxyl sulfate, 4-HPA: 4-hydroxyphenylacetate, DMA: dimethylamine, PAG: phenylacetylglutamine, p-CS: p-cresol sulfate, Pro-Bet: proline betaine.

A PCA of identified metabolites showed clear time-of-day variation in the first principal component (PC1), with high mean PC1 scores in the morning and low mean PC1 scores in the evening (Fig. 4). The PC1 vs PC2 scores matrix is shown in Supplementary Fig. S2.

**Figure 5:**
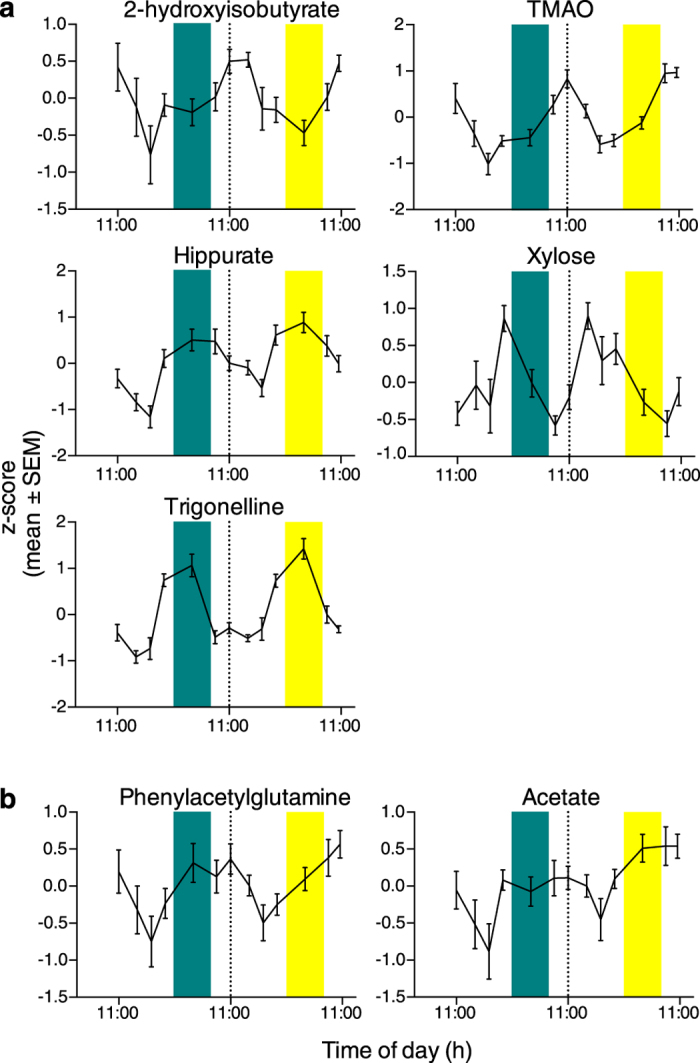
Urinary metabolites with significant 24 h cosine rhythms. (**a**) Metabolites exhibiting a significant 24 h cosine rhythm on both day 1 (wake/sleep) and day 2 (wake/wake). (**b**) Metabolites with a significant cosine rhythm on day 2 only. The midpoint of each urine collection interval was used for the analyses. Dashed line shows day 1/day 2 boundary. Green bars show sleep period (23:00–07:00 h); yellow bars show sleep deprivation period (23:00–07:00 h).

Of the 32 identified metabolites, seven (22%) exhibited a significant cosine rhythm on at least one of the 24 h days (Fig. 5). Of these, five (2-hydroxyisobutyrate, TMAO, hippurate, xylose and trigonelline) exhibited a cosine rhythm on both days, and two (acetate and phenylacetylglutamine) had no rhythm on day 1, but exhibited a rhythm on day 2. Three of the significantly rhythmic metabolites peaked during the day: 2-hydroxyisobutyrate (12:02 h day 1; 12:42 h day 2); TMAO (10:06 h day 1; 09:02 h day 2) and phenylacetylglutamine (10:21 h day 2), while the remaining four metabolites peaked during the night (19:56–03:58 h day 1; 18:14–04:44 h day 2). Of the five metabolites that exhibited a cosine rhythm on both days, 3 had an increase in amplitude during the 24 h of continued wakefulness on day 2: 2-hydroxyisobutyrate (17.5% increase); TMAO (18.6%) and trigonelline (15.5%). Hippurate and xylose both had no change in amplitude on day 2 (small < 0.1% decrease).

### Inter-metabolite correlations

**Figure 6:**
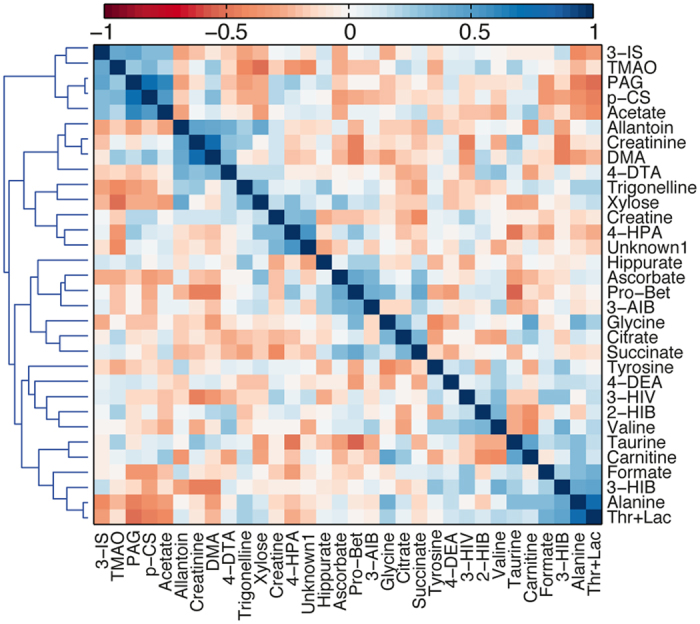
Correlations between urine metabolites measured in the study. Hierarchically clustered heatmap showing the Spearman’s rank sum correlation between the identified urinary metabolites. 2-HIB: 2-hydroxyisobutyrate, 3-AIB: 3-aminoisobutyrate, 3-HIV: 3-hydroxyisovalerate, 3-IS: 3-indoxyl sulfate, 4-HPA: 4-hydroxyphenylacetate, DMA: dimethylamine, PAG: phenylacetylglutamine, p-CS: p-cresol sulfate, Pro-Bet: proline betaine.

Figure 6 shows the Spearman correlations between the different metabolites based on their concentration levels at all time-points using data from all study participants. 103 metabolite pairs (out of a total 496 possible pairs) have significant positive correlations, while 137 metabolite pairs have significant negative correlations (q ≤ 0.05). The following metabolite pairs have significant Spearman’s rank correlation ρ > 0.5: Thr+Lac and alanine (ρ = 0.78), dimethylamine and creatinine (ρ = 0.76), p-cresol sulfate and phenylacetylglutamine (ρ = 0.73), acetate and phenylacetylglutamine (ρ = 0.62), Unknown 1 and 4-hydroxyphenylacetate (ρ = 0.54), and acetate and p-cresol sulfate (ρ = 0.53). The following metabolite pairs have significant Spearman’s rank correlation ρ < −0.5: proline betaine and taurine (ρ = −0.55), TMAO and xylose (ρ = −0.52), and Thr+Lac and phenylacetylglutamine (ρ = −0.51).

## Discussion

In this study, we looked at the effect of a night of acute total sleep deprivation (23:00–07:00 h) on the human urine metabolome, and found 50% of the identified metabolites with significantly changed levels compared to a night of sleep. Four metabolites were still significantly different in the morning (07:00–09:00 h) following a night of sleep compared with a night of total sleep deprivation while at later time points no significant differences were observed in any metabolite levels. The diurnal, time-of-day rhythms of the metabolites under strictly controlled conditions were also examined, with 22% of the identified metabolites exhibiting a significant 24 h cosine rhythm on at least one 24 h day.

3-indoxyl sulfate was one of the metabolites with the largest average change during a night of sleep deprivation (23:00–07:00 h: 43.7% increase), and 3-indoxyl sulfate levels were still significantly increased the following morning (07:00–09:00 h: 29.8% increase). 3-indoxyl sulfate is a metabolite of dietary tryptophan. Intestinal bacteria metabolize tryptophan to an indole, which is further metabolized to 3- indoxyl sulfate in the liver before being excreted into the urine^27^. The increase of 3-indoxyl sulfate in urine during and after sleep deprivation observed in this study is unlikely to result from dietary sources as the study participants were given the same standardized meals at identical times on both days, thus the increased levels are assumed to reflect differences in metabolism during sleep and sleep deprivation. Another metabolite of tryptophan, serotonin, plays a major role in sleep/wake regulation, primarily by promoting wakefulness and inhibiting rapid eye movement (REM) sleep^28^. Tryptophan and serotonin could not be quantified from the urine metabolic profiles due to low concentrations and large overlap in the spectral regions. However, both tryptophan and serotonin were increased during sleep deprivation in plasma samples obtained from the same study participants^14^. Increased levels of 3-indoxyl sulfate observed in urine during and after sleep deprivation may thus reflect our observation of increased tryptophan and serotonin levels in plasma from the same study participants. Our findings of increased urinary 3-indoxyl sulfate and plasma tryptophan and serotonin^14^ during sleep deprivation are supported by a recent sleep restriction study^15^, together implicating a role for tryptophan/serotonin metabolism in sleep/wake processing.

In our previous publication on plasma metabolites during sleep deprivation^14^, 3-indoxyl sulfate was not quantified. However, a study by Dallmann *et al.*^29^ showed increased 3-indoxyl sulfate in plasma during 40 h of wakefulness. 3-indoxyl sulfate is a well-known uremic toxin accumulating in the blood of chronic kidney disease (CKD) patients due to inadequate renal clearance^30^,^31^, and has been associated with increased risk of CVD in these patients^32^,^33^, possibly through increased oxidative stress^34^,^35^. Whether increased levels of 3-indoxyl sulfate might be involved in the increased risk of CVD linked to sleep deprivation merits further investigation.

Of the urine metabolites identified in this study formate had the largest mean increase (45.6%) during sleep deprivation. Formate is the simplest of the carboxylic acids, and an intermediate of several metabolic processes. Increased formate levels during sleep deprivation might reflect higher metabolic rates in general during wakefulness compared to during sleep^36^,^37^. Citrate levels were also significantly increased during sleep deprivation, and increased levels of citrate and formate may reflect a change in the transport of these acids in the kidney.

Carnitine had the third largest mean change (+31.0%) in this study. Carnitine is a non-essential amino acid which is an essential factor in fatty acid metabolism in mammals. Acylcarnitines are formed for transport of fatty acids into the inner mitochondrial membrane by conjugation of long-chain fatty acids to carnitine, where inside the mitochondria the fatty acids are catabolized to generate acetyl-CoA, NADH, and FADH_2_. In support of the urine data, we did observe increased levels of several acylcarnitines in plasma samples from the same subjects^14^, suggesting changes in β-oxidation of fatty acids during sleep deprivation.

The urine metabolome showed large time-of-day variations as evidenced by PCA of the metabolite levels. The mean PC1 score was at its highest during the morning (07:00–09:00 h collection interval), with PC1 loadings showing high levels of phenylacetylglutamine, p-cresol sulfate, acetate and 3-indoxyl sulfate. By contrast, the mean PC1 score was at its lowest during the evening (19:00–23:00 h) with high levels of alanine, allantoin, trigonelline, thr+lac, formate, xylose, valine, and tyrosine, among others. These metabolites represent the metabolites exhibiting the largest variation across time, possible as a result of food intake, activity levels, sleep/wake and light/dark differences. There was no apparent difference between the sleep deprived night and the nights of sleep in the PCA, thus the time-of-day variation in urine metabolite levels can be assumed to be larger than the metabolic changes observed during sleep deprivation. Seven urine metabolites exhibited a significant cosine curve, either on one or both days of the study, highlighting the importance of time of sampling in urine metabolomics studies. The timing of a 24 hour urine collection (start and end time) would also affect metabolite levels. Moreover, since individuals differ in circadian phase and sleep timing (e.g. morning and evening types), standardizing start and end 24 hour urine collection times based on clock time, rather than sleep start and end times, would not take account of individual differences in circadian and sleep timing.

Creatinine levels are frequently used for normalization of urine metabolomics data, with the assumption of creatinine expression being relatively constant over time. Our results however show diurnal variations in creatinine levels during the 24 h day, and during sleep deprivation compared to during sleep, suggesting that creatinine normalization may not be a good choice for studies based on spot urine collection as diurnal variation in the urine levels is to be expected. Time of day should, therefore, be taken into account when comparing results of spot urines with normal ranges.

There was a confounding effect of the protocol on taurine levels, with levels increasing over the study period. This was not the case for plasma taurine levels from the same subjects, with no significant effect of the protocol observed^14^. Increased taurine levels during the in-laboratory session might result from inactivity during the protocol. Urinary taurine levels were, however, significantly different during sleep deprivation compared to during sleep, both with and without correcting for the increase of taurine levels over time. As previously discussed^14^, increased taurine in plasma and urine may be involved in the regulation of sleep/wake transitions^38^,^39^ and with depression^40^,^41^.

Measuring metabolite levels over time presents a useful opportunity to examine how the metabolites co-vary. Our data show that the excretion of several urine metabolites were correlated. The metabolites 3-indoxyl sulfate, TMAO, phenylacetylglutamine, p-cresol sulfate, and acetate are positively correlated and cluster together. They share common ground in being dietary metabolites; 3-indoxyl sulfate, TMAO, and p-cresol sulfate result from microbial degradation of dietary tryptophan, choline, and tyrosine, respectively^27^,^42^. Phenylacetylamine levels have been shown to positively correlate with vegetable intake^43^, while acetate is a metabolite synthesized during gut microbial degradation of food. Interestingly, some of these typical canonical gut microbial derived metabolites (acetate, TMAO, and 3-indoxyl sulfate) were significantly affected by sleep deprivation. By contrast alanine and lactate, which are strongly correlated to each other (presumably due to the Cori or alanine cycle) and negatively correlated to the gut-microbial derived set, exhibited no effect upon sleep deprivation. Given that food intake was regular and highly controlled in this study, we conclude that variation in dietary intake alone does not explain the systemic variation in the levels of these metabolites, even those derived primarily by gut microbes from dietary input.

Samples harvested at different times of day might show variations related to external factors such as circadian rhythms, food intake, and the patient’s sleep status, masking the relevant metabolic changes resulting from the disease or drug under study. Several metabolomics studies describe associations between the urine metabolites described in this study and health outcome^2^,^3^,^24^. Our results show that assessing the bias in time of sampling and/or modelling this variation might be critical in order to interpret associations more accurately, and small, but relevant metabolic changes might be masked by the larger time-of-day variations present in the data.

The urine metabolites as measured by NMR spectroscopy show significant time-of-day variation, with 22% of the identified metabolites exhibiting a significant 24 h cosine rhythm. Thus, the time of sampling might be highly relevant for biomarker discovery in urine metabolomics studies and accurate interpretation of spot urine collections. In addition, we report novel effects of sleep deprivation on several urinary metabolites, particularly formate, 3-indoxyl sulfate, carnitine and taurine. Improving our understanding of how the metabolome is influenced by circadian rhythms, time of day variation, and sleep patterns is critical for a more accurate interpretation of the relationships between the exposome, metabolome and risk of chronic disease.

## Additional Information

**How to cite this article**: Giskeødegård, G. F. *et al.* Diurnal rhythms in the human urine metabolome during sleep and total sleep deprivation. *Sci. Rep.* **5**, 14843; doi: 10.1038/srep14843 (2015).

## Supplementary Material

Supplementary Information

## Figures and Tables

**Table 1:** Changes in metabolite levels during and following 8 hours of total sleep deprivation.

Metabolite	Sleep night vs sleep deprivationnight (23:00–07:00 h)			First morning collection(07:00–09:00 h) after sleep andsleep deprivation night		
	% meanchange	p value	q value	% meanchange	p value	q value
Dimethylamine	−16.4	6.10·10^−6^*	2.00·10^−4^*	−4.76	0.0211*	0.0855
4-DTA	−14.3	1.31·10^−5^*	2.00·10^−4^*	−8.05	0.00182*	0.0328*
Taurine	25.3	1.85·10^−5^*,^a^	2.00·10^−4^*	14.3	0.00321*,^b^	0.0328*
Creatinine	−12.3	5.68·10^−5^*	4.00·10^−4^*	−4.90	0.244	0.375
Formate	45.6	6.95·10^−5^*	4.00·10^−4^*	11.8	0.276	0.402
Citrate	19.3	1.29·10^−4^*	7.00·10^−4^*	5.73	0.0325*	0.110
3-indoxyl sulfate	43.7	5.67·10^−4^*	0.00260*	29.8	0.00411*	0.0328*
Ascorbate	−22.4	7.69·10^−4^*	0.00310*	1.50	0.857	0.914
2-hydroxyisobutyrate	−9.32	0.00115*	0.00410*	−5.89	0.0744	0.170
Allantoin	−9.05	0.00132*	0.00420*	−8.44	0.0930	0.198
Carnitine	31.0	0.00180*	0.00520*	−1.88	0.896	0.914
3-hydroxyisobutyrate	10.6	0.00223*	0.00590*	0.74	0.729	0.864
4-DEA	−9.19	0.00251*	0.00620*	−5.51	0.0557	0.137
TMAO	10.6	0.00339*	0.00770*	13.0	0.0389*	0.113
4-hydroxyphenylacetate	−7.69	0.00391*	0.00830*	−9.28	0.0214*	0.0855
Acetate	9.30	0.0100*	0.0201*	6.98	0.140	0.248
Proline betaine	3.16	0.0374*	0.0705	−3.44	0.858	0.914
Phenylacetylglutamine	−14.0	0.0496*	0.0830	8.69	0.914	0.914
3-aminoisobutyrate	6.27	0.052	0.0830	5.74	0.116	0.218
p-cresol sulfate	−13.6	0.051	0.0830	−1.32	0.561	0.748
Succinate^c^	9.41	0.058	0.0884	−2.19	0.668	0.823
Xylose	−9.31	0.072	0.104	0.257	0.892	0.914
Unknown1 (d, 7.8 ppm)	−12.3	0.081	0.111	−2.63	0.103	0.205
3-hydroxyisovalerate	1.48	0.083	0.111	−0.40	0.316	0.440
Glycine	9.39	0.113	0.145	−17.1	0.00389*	0.0328*
Tyrosine	−1.24	0.123	0.151	−5.55	0.00689*	0.0441*
Creatine	−7.40	0.130	0.154	1.25	0.611	0.782
Trigonelline	17.9	0.242	0.277	14.9	0.225	0.375
Hippurate	−1.21	0.314	0.335	−11.1	0.246	0.375
Valine	−2.37	0.306	0.335	−2.21	0.0344*	0.110
Thr+lac	1.41	0.611	0.631	−10.7	0.0456*	0.122
Alanine	1.70	0.784	0.784	−7.72	0.0163*	0.0855

Negative differences represent decreased mean levels during sleep deprivation/the morning following sleep deprivation. The reported p-values are from linear mixed models analysis and the reported q-values are after adjusting for multiple testing using the Benjamini-Hochberg correction. d: doublet, Thr+lac: doublet containing signal from both threonine and lactate (1.33 ppm).

^a^After correcting for the confounding effect of taurine, differences were still significant (p = 0.0082, q = 0.0175).

^b^After correcting for the confounding effect of taurine, differences in taurine levels were not significant (p = 0.2274, q = 0.3751).

^c^Singlet at 2.4 ppm assumed, but not verified by standard addition, to be succinate, *p value/q value < 0.05.
