# Supplementary material for: Diurnal rhythms in the human urine metabolome during sleep and total sleep deprivation
Source: Sci Rep. 2015 Oct 9;5:14843. doi: 10.1038/srep14843 (PMC4598809; doi:10.1038/srep14843)
Supplement: Supplementary Information [file srep14843-s1.doc]

**Supplementary Information: Diurnal rhythms in the human urine metabolome during sleep and total sleep deprivation**

Guro F. Giskeødegård, Sarah K. Davies, Victoria L. Revell, Hector Keun, Debra J. Skene


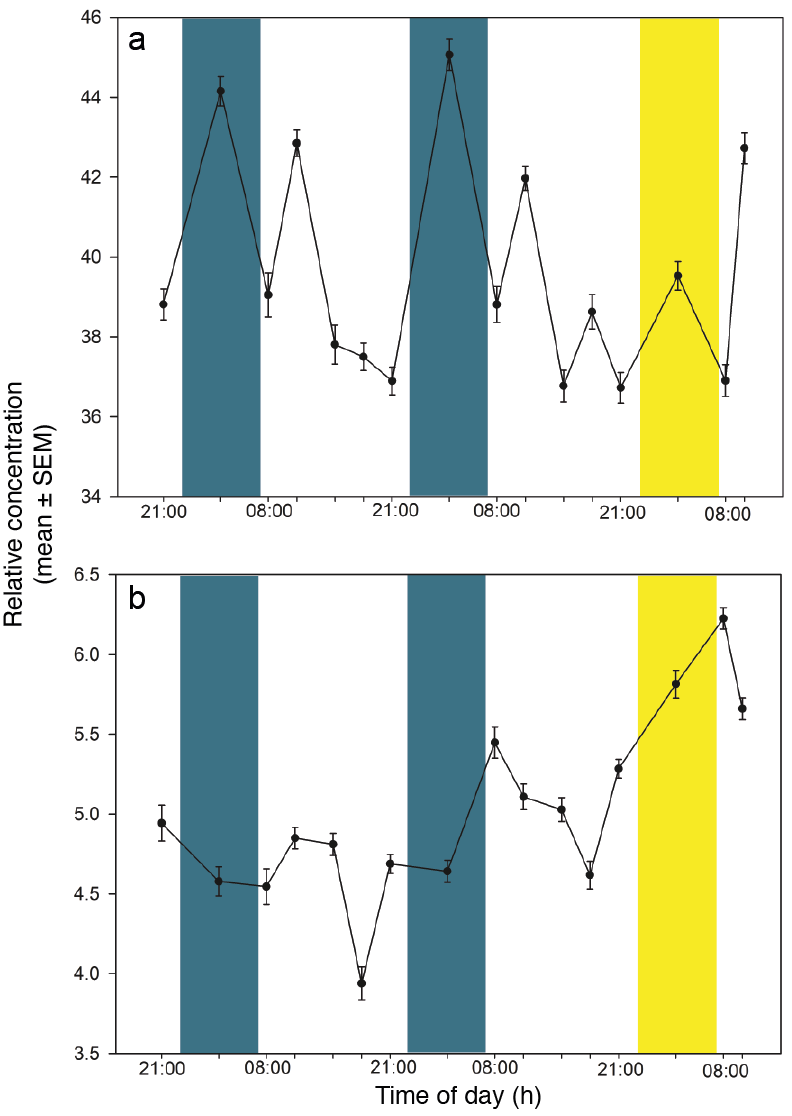


**Supplementary Figure S1. Urinary levels of creatinine and taurine during the study.** Mean (± SEM) creatinine (a) and taurine (b) levels (across all 15 individuals) plotted against time. The individual time points are the midpoint times from intervals of pooled urine samples obtained sequentially across the 60 h study protocol. Green areas: sleep period, yellow area: sleep deprivation night.

**Supplementary Figure S2. Principal component analysis (PCA) of all samples**

**and all metabolites (n = 32): PC1 vs PC2.** Samples are coloured by time point.
